# Between hope and future planning: the dementia journey for care partners through the lens of relational autonomy

**DOI:** 10.1186/s12910-025-01197-2

**Published:** 2025-03-21

**Authors:** Alixe Ménard, Adebusola Adekoya, Elizabeth Birchall, Kishore Seetharaman, Lucy Kervin, Koushambhi Khan, Jennifer Baumbusch

**Affiliations:** 1 Faculty of Health Sciences, Interdisciplinary School of Health Sciences, University of Ottawa, Ottawa, ON Canada; 2 School of Public Health Sciences, University of Waterloo, Waterloo, ON Canada; 3 School of Social Work, McMaster University, Hamilton, ON Canada; 4 Department of Gerontology, Simon Fraser University, Burnaby, BC Canada; 5 School of Nursing, Faculty of Applied Science, University of British Columbia, T201 2211 Wesbrook Mall, Canada BC V6T2B5 Vancouver,

**Keywords:** Dementia, Caregivers, Advance care planning, Relational autonomy

## Abstract

**Background:**

Future planning is essential for care partners to discuss and prepare for the goals of care for their relatives living with dementia. However, engaging in these discussions can be particularly challenging as care partners navigate the unpredictable and uncertain trajectory of dementia. This study aimed to explore how care partners of persons living with dementia engage in future planning (or not) throughout the dementia journey.

**Methods:**

This multi-method qualitative study used a relational autonomy framework to examine the experiences of care partners providing daily care to a person living with dementia. Fifteen care partners from British Columbia, Canada, participated in semi-structured interviews and maintained reflective diaries over a period of up to two years (August 2020–October 2023). Data were analyzed using thematic analysis to identify patterns and themes related to future planning.

**Results:**

Four key themes were identified through the analysis: (1) changes to living arrangements, as care partners adjusted to the evolving needs of their relatives; (2) anticipatory grief, reflecting the emotional impact of witnessing the progression of dementia; (3) future planning with changing health, highlighting the challenges of aligning care plans with the shifting health status of the person living with dementia; and (4) finding hope, as care partners sought meaning and optimism amidst uncertainty.

**Conclusions:**

This study underscores the complex and dynamic nature of future planning for care partners of individuals with progressive dementias. The findings highlight the need for tailored resources and interventions to support care partners in navigating future planning discussions, particularly in light of the emotional and relational challenges they face. Developing such resources could improve the preparedness and well-being of care partners as they engage in this critical aspect of caregiving.

**Supplementary Information:**

The online version contains supplementary material available at 10.1186/s12910-025-01197-2.

## Background

Future planning is a broad approach to anticipatory planning that offers people an opportunity to consider a wide range of future decisions rooted in their personal preferences, across legal, financial, and personal domains [1]. Future planning and advance care planning are related but distinct forms of anticipatory decision-making that support persons living with dementia and their care partners in preparing for future needs. Advance care planning is a subset of future planning focused specifically on healthcare decisions, designed to address the unique medical needs and preferences of persons living with dementia [2]. It is defined as “[enabling] individuals to define goals and preferences for future medical treatment and care, to discuss these goals and preferences with family and health-care providers, and to record and review these preferences if appropriate” ([2], p. e546]).

In summary, future planning encompasses a spectrum of life planning matters, while advance care planning narrows its focus specifically to healthcare decisions. Both forms of planning are essential for maintaining autonomy and engagement in decision-making for individuals living with dementia and offer support for care partners, reducing the decision-making burden and preparing them for the progression of dementia [3]. This study uses future planning terminology to acknowledge the comprehensive needs and preferences of both persons living with dementia and their care partners beyond healthcare, while recognizing advance care planning as a healthcare-specific component within this broader planning process. Although the concept of future planning is central to this study, there is a paucity of literature specifically addressing future planning for persons living with dementia and their care partners. As a result, references to advance care planning literature have been included to support the discussion, given the thematic overlaps between advance care planning and future planning in the context of decision-making and preparedness for future needs.

Future planning can be important for persons living with dementia to maintain autonomy and engagement in decision-making as their disease progresses, and for care partners to discuss goals of care for their relatives [1]. Care partners are nonprofessional and/or unpaid individuals (e.g., spouses, siblings, children, other relatives, or friends and neighbours), who assume primary responsibility for providing assistance to someone who requires ongoing support with activities of daily living due to a chronic illness or disability in managing their health [4]. For care partners, future planning for the person living with dementia involves conversations about goals of care, appointing substitute decision-makers, deciding on the place of death, considering the potential move to a long-term care or nursing home, and establishing preferences for their care as dementia progresses [5]. As the person living with dementia loses functional and cognitive abilities or decision-making capacity, future planning can help reduce decisional burden in care partners and increase acceptance surrounding the prospect of the person’s decline and eventual death [6]. Making care-related decisions can be extremely stressful for care partners and a contributor to the burden of care [7].

Current literature from the United States, Canada, United Kingdom, Western and Northern Europe, Australia, and New Zealand suggests there is limited engagement in future planning for dementia among persons living with this disease and their care partners, or healthcare providers, despite evidence highlighting its critical necessity within this demographic [3, 8]. Recent reviews also indicate limited research on the facilitators and barriers to future planning for individuals with neurodegenerative disorders such as dementia and their support networks [9, 10]. Extant studies suggest that barriers to future planning among persons living with dementia and their care partners include: limited knowledge and awareness of future planning, challenges in finding the appropriate timing for conversations, favouring informal plans over written documentation, restrictions on choices regarding future care, and insufficient support from health and social care providers for making decisions about future healthcare [11]. Research on obstacles to future planning among persons with chronic and/or life-limiting illness indicates additional barriers related to finances, lack of information, lack of services, and resistance from family members to engage in future planning discussions stemming from difficulty understanding and accepting a new future with the illness [1] and negative associations of future planning with death and hopelessness [12].

Against this backdrop, this study aims to address the research question: How do care partners of persons living with dementia engage in future planning (or not) during the unpredictable/uncertain dementia journey? By conducting semi-structured interviews and collecting diaries over a span of two years, the research delves into the dynamic experiences and obstacles encountered by care partners throughout the dementia journey. Embracing the framework of relational autonomy, the study seeks to illuminate the nuanced ways in which care partners engage in future planning.

## Theoretical Perspective

We used relational autonomy as a theoretical lens to explore the future planning experiences of care partners of persons living with dementia. Relational autonomy conceptualizes autonomy within the social and relational contexts in which a person is situated [13] and is associated with the ethics of care theory which values care relationships and interdependency [14]. Ethics of care has emerged in a small body of work in dementia research and refers to the qualities necessary for care partners to provide compassionate and effective care for individuals in need of support [15]. It also highlights the political and societal commitment required to ensure that both care partners and care recipients receive the support needed for caregiving to be carried out with dignity and respect [15, 16].

In the context of relational autonomy, actions, decisions, values, beliefs, and identities are understood as being influenced by one’s relationships with others [14]. This approach adopts a personhood lens, emphasizing that people living with dementia are more than their neurological deficits; they are seen as whole persons, shaped by their unique histories, experiences, and relationships [17]. The importance of assessing people’s needs and rights within their social relationships, and how these relationships can foster or undermine their agency, is emphasized [18], and decision-making processes are acknowledged as inseparable from social environments or culture [19]. Relational autonomy provides a framework for understanding decision-making as embedded within social contexts and interactions and promoting autonomy for individuals with cognitive impairments who depend on the support of others [18, 20]. Exploring future planning through the lens of relational autonomy promotes analysis and interpretation that is attentive to the complexities of interpersonal relationships and the interconnection between individual agency and social support.

## Methods

### Study aim, design and setting

Data were drawn from a multi-year, multi-method qualitative research study investigating the experiences of community-dwelling persons living with dementia and their family care partners navigating formal services and supports. We chose to investigate the topic of future planning given the recurring occurrence of this phenomenon in the data, warranting further analysis.

The setting for this study was British Columbia in Western Canada; this province has among the fastest projected rates of growth of dementia onset across all Canadian regions [21]. This study was, unexpectedly, conducted in the midst of the Coronavirus (COVID-19) pandemic [22]; however, our intention was to explore care partners’ experiences broadly rather than those specific to the pandemic context. The study received research ethics approval from the University of British Columbia’s Behavioural Research Ethics Board.

### Participants and recruitment

A purposive criterion-based sampling method was used [23]. Care partners of persons living with dementia were recruited through advertisement in community organization newsletters, social media advertisement, and word-of-mouth referral. To be eligible for participation, care partners had to meet the following criteria: (a) primary provider of care for a community-dwelling person living with dementia, (b) living in British Columbia, Canada, and (c) fluent in English. Participants were screened for eligibility by a research team member following initial contact.

### Data collection

Data were collected over a two-year period (August 2020 to October 2023) via semi-structured interviews and written diary reflections. The interview guide used to collect data presented in this paper (Supplementary File 1) was implemented as part of the overarching research project investigating the experiences of families navigating care in the community, which has been described in additional detail elsewhere [22]. Diary entries revealed the spontaneous, emotional, and immediate aspects of planning, while interviews provided a space for retrospective reflection, enabling a deeper exploration of how relationships influenced decision-making over time. Combining diary entries and interviews allowed insights into care partners’ thoughts, emotions, and experiences as they naturally unfolded over time [24], offering rich, nuanced insights into day-to-day caregiving decisions and future planning.

Informed consent was obtained from participants prior to all data collection. Interviews were performed every four months by video call (Zoom) or telephone, ranged in duration from 33 to 155 min, and were recorded and transcribed. Reflexive notes were taken during or immediately following the interview to support data analysis and interpretation. Diary completion was flexible and accommodated participants’ preferences and availability but was generally performed monthly. Data collection was performed by two research team members (KS & HC) with knowledge of dementia care and graduate training in qualitative methods, and continued until either two years of data were collected or until the death or transition of the person living with dementia into a long-term care home. Participants completed between two and six interviews, with the frequency depending on the stage of their caregiving journey (e.g., concluding after their relative with dementia passed away). Additionally, participants contributed between 5 and 20 diary entries, depending on their individual inclination to document their experiences. Identifying information was removed from interview transcripts and diary entries and participants were assigned codes to preserve anonymity. Interviews and diary reflections explored the family’s dementia journey, daily routines, supports, and overall wellbeing.

### Data analysis

Management of transcripts, diary entries, and reflexive notes was facilitated using qualitative analysis software NVivo 12. Interview and diary data were analyzed using reflexive thematic analysis [25]. Reflexive thematic analysis is an interpretative approach that acknowledges how the meaning derived from data is profoundly influenced by factors such as the researcher’s perspective, the study context, and the social and relational dynamics embedded within the data [26]. This approach aligns with the theoretical perspective of relational autonomy as, through its application, resulting themes serve as tangible reflections of the social and relational contexts inherent to future planning. In alignment with reflexive thematic analysis, we established patterns of meaning by recursively engaging with the data [25, 27]. Transcripts and diary entries were reviewed closely to identify excerpts that were explicitly or implicitly concerned with future planning. Guided by the concept of relational autonomy, data were coded deductively, with attention to the research question and the dynamics of interdependence and mutual recognition in care relationships. We systematically reviewed interview transcripts to identify explicit or implicit references to future planning, including advance care planning, end-of-life decision-making, and discussions about long-term care needs. Excerpts were categorized under key tenets of relational autonomy, such as the influence of social contexts, negotiated decision-making, and the role of care networks [19]. This process involved intentionally seeking and extracting excerpts where care partners mentioned concerns, preferences, or decisions related to future planning.

During the initial coding phase, excerpts were systematically assigned to pre-defined codes reflecting relational autonomy by the full author team (AM, AA, KS, LK, KK & JB), including family experiences, changes in care roles, access to support systems, evolving living arrangements, and emotional and logistical aspects of end-of-life planning. These codes captured how future planning was negotiated within relationships**,** highlighting interdependence and shared decision-making.

Following initial coding, the full author team (AM, AA, KS, LK, KK & JB) engaged in iterative discussions to ensure consistency in code application based on evolving nuances. Codes were reflexively reviewed and refined in regular meetings, and as patterns were identified, related codes were grouped into broader thematic categories. This process ensured that themes accurately captured shared experiences and perspectives across participants. Ultimately, the analysis yielded four overarching themes that reflected similarities in meaning and experiences across participants.

### Study rigor

Rigour was established through processes related to study credibility, transferability, dependability, and confirmability [28]. Credibility (i.e., presentation and interpretation of participants’ experiences that is close to their reality and recognizable to others sharing similar experiences) was established through research team reflexivity (e.g., reflexive notes), debriefing among team members at meetings during data collection and analysis, and use of participants’ own words in thematic development. Transferability (i.e., supporting the applicability of the knowledge developed to other contexts or settings) was promoted by purposive sampling and efforts to represent a range of familial dynamics and relationships. Dependability (i.e., development of a comprehensive decision trail so the findings of the study could be repeated given the same cohort, coders, and context) was established through use of interview and diary guides, application of a study protocol, and detailed record-keeping of the data collection process. Confirmability (i.e., enhancing the likelihood that findings would be corroborated by other researchers) was ensured by triangulating data from multiple sources, open-ended questioning, and proactive requests from participants for clarification as needed.

## Results

Recruitment and screening yielded a sample of fifteen care partners. The majority of participants provided care for their spouse (*n* = 6) or parent (*n* = 7); others cared for a sibling (*n* = 1) or mother-in-law (*n* = 1). There was a higher proportion of woman care partners (*n* = 12) than men (*n* = 3). Participants were aged between 36 and 83 years and cared for persons living with dementia whose age ranged from 62 to 101 years. Many participants were university-educated (40%) and most were retired (73%). The majority of participants were White (93%) and over half had an annual household income over $61,000 (67%). Table 1 details the characteristics of participants.

**Table 1:** Participant demographics

Descriptive characteristics	Total sample (*N* = 15)	%
Age (years)		
Mean	63.5	
Range	36–83	
Gender		
Female	12	80.0
Male	3	20.0
Education level		
High school diploma	3	20.0
College diploma	6	40.0
University degree	6	40.0
Race		
White	13	86.7
South Asian	1	6.7
Mixed Race	1	6.7
Relation to person living with dementia		
Spouse	6	40.0
Daughter	7	46.7
Daughter-in-law	1	6.7
Sibling	1	6.7
Employment status		
Full-time	2	13.3
Part-time	1	6.7
Retired	11	73.3
On leave	1	6.7
Annual income (household)		
< $20,000	1	6.7
$41,000-$60,000	3	20.0
$61,000-$80,000	5	33.3
$81,000-$100,000	1	6.7
> $101,000	4	26.7
Prefer not to say	1	6.7
Language primarily spoken at home		
English	14	93.3
Other	1	6.7
Number of people living in household		
Mean	3	
Range	1–7	
Age (years) of persons living with dementia		
Mean	82.6	
Range	62–101	
Gender of person living with dementia		
Female	9	60.0
Male	6	40.0
Number of completed interviews		
2	2	13.3
3	1	6.7
4	1	6.7
5	5	33.3
6	6	40.0
Number of completed diaries		
0–4	3	20.0
5–8	2	13.3
9–12	3	20.0
13–16	4	26.7
17–20	3	20.0

The patterns of future planning based on participants' narratives are presented in four key themes: (1) changes to living arrangements; (2) anticipatory grief; (3) future planning with changing health; and (4) finding hope. Participants highlighted various facilitators and barriers to engaging in future planning, underscoring the critical importance of interdependence between persons living with dementia and their care partners. They also emphasized decision-making within care networks and a deep respect for the personhood of individuals living with dementia.

### Changes to living arrangements

The theme *Changes to Living Arrangements* showcases care partners’ adaptive responses to their relatives’ changing needs. Many care partners strive to preserve their relatives' dignity, cultural identity, and quality of life amidst logistical and ethical dilemmas, highlighting the intertwined nature of emotion and caregiving. This theme illustrates the proactive residential adjustments made by care partners in response to the evolving needs of their relative living with dementia. Home modifications included: putting their dining room table in storage to accommodate a bed (P01); moving their bedroom to the basement to allow their relative to sleep in the bedroom on the main floor (P03); and buying a bigger home to make room for their relative living with dementia (P14).

Another type of residential shift occurred when a relative living with dementia moved into long-term care (LTC). In this instance, the care partner (P08) acknowledged the severity of their relative’s dementia symptoms and took the proactive step of initiating the LTC placement process by placing them on a waitlist. Conversely, in the case of P10 and her mother whom she cared for, P10A, the decision-making process took a different route. Originally, P10A was in an assisted living arrangement with her husband. After his passing and as her dementia progressed, P10A’s children considered moving her to a care home. However, P10, influenced by previous negative experiences with the same care home, chose instead to bring her mother into her own home. It was only after P10A moved in with her that P10 fully realized the severity of her mother’s dementia. This scenario underscores the complexity and deeply personal nature of decisions regarding the care of relatives with dementia, reflecting how past experiences and the immediate realities of care needs can shape choices about living arrangements:

*They wanted to move her into long-term care at the facility that I had just spent a year trying to get my husband out of. And so I opted to bring her home…she’d been visiting me here for thirty years. I figured we could do it. And as soon as I brought her home and I realized the level of dementia – because you don’t see it if you’re living outside of it, I think, as much. At once, I contacted [health authority] right away. Initially I contacted them to put her on the list for the day program and I called back the next day and said, “Nope, she needs to be on the list for a care home.”* (P10, daughter, interview).

Changes to living arrangements also involved collaborative decisions among siblings who provided care. Such shared decision-making processes within the context of familial relationships and networks are in alignment with tenets of relational autonomy emphasizing interdependency and social influence. One participant discussed making changes to living arrangements to accommodate the needs of their relative and help them remain at home, which also led to benefits of sharing of care responsibilities and lowering housing costs for the care partners.

*My mum expressed that having my sister live with her would alleviate her loneliness, she could help with household chores and cooking, and that she would not have to, later on, if necessary, move into a “care home.” In turn, my sister would not face the current financial strain that has been her burden since her husband passed away…This move would also be helpful for me.* (P14, daughter, diary entry)

Decisions regarding placing or not placing their relatives in a LTC home were challenging for some participants, especially during the COVID-19 pandemic due to visiting restrictions and concerns about “poor care standards” in LTC homes. As P12 went on to write in her diary entry:

*But we still cannot bring ourselves to placing mom in care especially during this pandemic. We could not handle not being able to see mom if she was in a care home and there was a lockdown, and we could not visit. I've also read so many horror stories of the level of care provided in care homes from groups I belong to on Facebook (private groups) with many members whose parents or spouse is in a care home. Abuse, neglect, poor care standards… the list goes on*. (P12, daughter, diary entry)

Respect for personhood, a core concept of relational autonomy, was reflected in care partners efforts to respect their relatives’ wishes not to move to LTC. For P03B’s family, the fear related to LTC also included the loss of cultural identity:

*The East Indian culture is very…it’s quite family oriented, in a lot of good ways and a lot of not good ways [chuckling]. But it’s always multi-generational, a lot of people living in the house, cousins and uncles and things like that. (…) I knew it would kill my dad to go into a home. There’s no…even now, I couldn’t do it*. (P03B, daughter, interview)

Other participants had a fundamental objection to the prevailing perception or practice of LTC homes. This perspective was expressed by P08: *….Because long-term care facilities shouldn’t be just warehouses for people waiting to die. (…) These new facilities that are going to be built, there should be some sort of programming around there that is meant to help with the individual’s quality of life and as I say, not just put them in wheelchairs and put them in front of the TV and watching some reality show. That’s not what it’s about.* (…). P08 further elaborated on the importance, and lack of ongoing support for persons living with dementia and their care partners in LTC:

*There are people that are going through this right now and don’t know where to go. Will the health facility continue to help and support them in some way or are they going to just be left alone until they scream and say “I need your help”*? (P08, brother, interview)

### Anticipatory grief

Anticipatory grief refers to the emotional distress experienced prior to a loss, commonly felt by individuals who anticipate the death of someone close to them [29]. The theme *Anticipatory Grief* underscores the emotional challenges faced by care partners as they navigate the complexities of future planning, particularly in the context of changes in the person living with dementia and the inevitability of their death. This emphasizes the entanglement of emotion work and care work, as care partners grapple with both the emotional impact of anticipatory grief and the practical responsibilities of end-of-life decision-making.

For some participants, grief began well before the actual loss of their relative living with dementia as they grappled with the progressive nature of the disease and the eventual need for more care. This grief acted as both a propeller and a barrier to future planning. For some care partners, anticipatory grief drove the urgency to make funeral arrangements and decisions about end-of-life care, as they felt compelled to prepare for their relative’s passing. Conversely, others experienced such uncertainty around the disease trajectory that they were unsure whether death was imminent. This ambiguity often led to emotional turmoil, where they were torn between bracing for loss and holding on to a sliver of hope. This tension resulted in a ‘freeze’ reaction, where the prospect of future planning felt overwhelming and premature, causing some to delay or avoid these preparations entirely.

The following diary entry illustrates the anticipatory nature of the grief experienced by a care partner. It highlights the stress, emotional turmoil, and practical preparations associated with the potential loss of a relative living with dementia. Anticipatory grief involves grappling with the reality of an impending loss, often leading to a range of emotions from hope to despair, and taking actions in response to the anticipated future:

*This month was challenging. Specifically the last 2 weeks when he did not eat for 5 days and his stool was black, a sign of blood from a stomach ulcer. I was pretty worried. I called our son to get home if possible as soon as he could. Last Sunday we had the palliative care nurse come by the house to see how he was doing. Fortunately by then he had started eating again so she will send a report to his doctor and say that he is not eligible for palliative care unless he gets worse again.* (P03, wife, diary entry)

Another contributor to the participants’ anticipatory grief was the emotional aspect of making preparations to live with their relative through the progression of their dementia. Most care partners reported lacking knowledge about the dementia disease trajectory and how best to prepare for their relative’s inevitable decline. When faced with the need to transition their relative to a long-term care home, one care partner discussed their fear of forgetting what their relative was like prior to their dementia diagnosis as a potential barrier to future planning:

*I don’t know why I feel so overwhelmed. And I was like, “oh, right, this is what people talk about”…like putting your loved one into care is a very overwhelming emotional experience. And I was just like, “I won’t be like that.” And I think I was just not prepared to feel the way that I did, and like didn’t even really kind of register. Part of my anxiety was forgetting…just forgetting what he was like*. (P07, spouse, interview)

Anticipatory grief was also expressed because of challenges faced by care partners in distinguishing between generalized future planning and funeral arrangements. Care partners reported finding it difficult to confront the reality of their relative’s declining health and eventual death, leading to a reluctance or avoidance of discussions related to funeral arrangements. This emotional complexity can blur the distinction between generalized future planning, which may feel more manageable to address, and funeral arrangements, which may provoke more intense feelings of grief and loss. Some participants expressed hesitancy and emotional distress in confronting the eventual death of their relative living with dementia*,* “*You never want to talk about that [the death of a spouse or partner]. You never want to face that. You never want to think about that. But in the back of your mind, you know it’s there*”. (P05, husband, interview).

The emotional and mental overlap between general future planning and funeral arrangements often led care partners to prioritize more immediate concerns related to their relative’s care and well-being (e.g., coordinating medical appointments, managing medications, and making changes to their living arrangements) and postpone conversations about certain aspects of future planning until later. This hampered their willingness to actively participate in comprehensive future planning initiatives. For instance, when asked about planning for the future and making decisions as a family, a care partner said “*We haven’t done any funeral planning yet. I don’t think it’s time to be talking about that*” (P08, brother, interview). Conversely, when asked about future planning in consideration of their relative’s worsening health and entrance into a palliative care program, another participant mentioned plans for a death doula (i.e., a trained professional who provides emotional, spiritual, and practical support to an individual and their families in the dying process [30] and their wish that every family preparing for the death of a relative with dementia could have this form of support (P06, daughter, interview). This illustrates that holistic future planning may be undertaken by some care partners through their anticipation of emotional and spiritual needs associated with the eventual death of their relative, and the importance of this form of support, in addition to the practical aspects of future planning (e.g., financial and legal preparation) and beyond traditional funeral arrangements.

### Future planning with changing health

This theme encompasses future planning concerns arising from changes in the health of care partners and their relatives living with dementia. Participants expressed apprehension regarding the potential impact on their relative if they, as care partners, were to become ill or injured, rendering them unable to provide their usual level of care. These apprehensions underscore the significant toll caregiving takes on care partners’ own health and wellness, shaping their outlook on the future of care for their relatives with dementia. For example, in her diary entry, P12 (daughter) wrote “*These days I'm starting to feel exhausted and bewildered and wonder how much longer my sister and I can go on as family care partners what it is going to do to our own health."* Participants also discussed experiencing a decline in their own health related to caregiving and burnout:

*I told the doctor how overwhelmed I was getting with caring for P07A. All the programs had been shut back down [due to COVID-19 public heatlh measures], I could not supply the stimulation he needs on a daily basis. I am struggling myself …I am losing a sense of myself…I cannot leave him alone. I need programs and activities to keep him busy and healthy, and me, I need breaks from him. I am at a point of despair.* (P07, wife, diary entry).

In this quote, P07 acknowledges their inability to leave their relative alone, indicating a sense of obligation and commitment to their care. This illustrates the relational aspect of autonomy, where one's sense of self is defined in relation to their caregiving role. However, P07’s plea for support through programs and activities for their relative reflects their recognition of the importance of maintaining the health and well-being of both them and their relative. This demonstrates a desire for autonomy within the caregiving relationship, as P07 seeks resources to alleviate their burden and provide respite.

Participants reflected on the evolving nature of their relative’s memory loss, the need to accept and adjust to their relatives’ behavioural (e.g., ‘wandering’) and cognitive (e.g., memory loss) changes while balancing the implications of these changes for their relative’s care and support needs and shifting independence. P13 and his wife live in assisted living where she receives dementia care. He described the changes in her behaviour and how she required more supervision. Despite his wife’s tendency to engage in wandering, P13 described her as “independent,” indicating a desire to respect her autonomy and freedom of movement. However, P13 also acknowledged the challenges and risks associated with wandering behavior, such as the potential for his wife to walk away from assisted living unattended and become lost or injured:

*It’s too much trouble to keep track of her because she has a tendency to wander around. People watch her walking around and will come and tell me she’s over there. So, she is independent, and we get down to the first floor and we’re doing something together. “I’m just going to go down here a bit.” And I don’t know whether she picked up a snack, talked to people, sat down. But once again the nurses on the staff come back and say, “Oh, she went outside.” And three times we’ve brought her back and just keep her in for a while.* (P13, husband, interview)

As health conditions evolve, the autonomy of the person living with dementia and their care partners is intertwined. Decisions about healthcare, treatment options, and LTC are shaped through ongoing negotiation and communication within the social relationships (e.g., care partner siblings and their relative with dementia). As care partners are faced with complex, rapidly changing health trajectories, they experience subsequent impacts on themselves and their relative living with dementia both in relation to their health and ability to provide care and plan for shifting care needs.

### Finding hope

This theme underscores the resilience of care partners and the satisfaction derived from providing care to their relatives with dementia, aligning closely with the broader concept of future planning. Despite the inherent challenges associated with caregiving and the complexities of future planning, some participants shared positive experiences of caring for their relatives and interacting with healthcare providers. The support received from relatives, friends, neighbors, and healthcare providers played a pivotal role in helping these participants navigate the challenges of caregiving, ultimately contributing to their positive experience of care and laying the groundwork for effective future planning: “*I want to do these things for my mom and to not feel stressed and enjoy the time that I am doing things with my mom, even if it is just going to an appointment, you know*?” (P14, daughter, interview).

Some participants found hope for the future by cherishing happy moments shared with their relatives, expressing satisfaction with their caregiving efforts. Care partners also expressed hope for their relatives with dementia to receive consistent support across all stages of the condition, driving them to seek out available services. This hope motivated the exploration of various care options and interventions, such as researching LTC homes, consulting healthcare professionals, and engaging in advance care planning. Positive interactions with LTC staff during end-of-life caregiving were also shared by one participant (P11, daughter, interview). The care partner began by reflecting on the meaningful interactions she observed between the staff and her mother, which contributed to a sense of dignity and humanity:

*[Staff] would come in and engage with me and it really made me feel like my mum was a human being that was worthy of not just medical care but as a human being who’s at the end of her life.*

Here, the care partner highlighted how compassionate care from the staff instilled hope, as it affirmed her mother’s worth beyond mere medical needs. This level of care allowed her to see that her mother was valued as a person, offering comfort and hope amid an emotionally difficult time. The daughter continued, contrasting her own experience with the often heartbreaking stories she had heard from others about loved ones receiving end-of-life care:

*And I know from talking to others and their sad stories about what they’ve gone through with a loved one dying in long term care, that’s not generally the kind of care that you get at end of life…*

This comparison revealed a deeper layer of hope, that compassionate and person-centered care might become more common for others in similar situations. Her experience stood out to her as exceptional, something that she recognized is not universally available, adding a sense of gratitude to her hope. Finally, she shared a sense of peace about her mother's end-of-life care, expressing a form of hope even in the midst of her loss:

*She has the best possible care that she could have. And I felt like because sometimes when things would get awful, I’d say, this can’t be how our story ends. And now I look back as heartsick as I feel that it’s over, I’m glad that’s how it ended with beautiful care and with her surrounded.*

This closing reflection captured hope in its fullest sense. The daughter found solace in knowing her mother’s life concluded in a compassionate, dignified environment, surrounded by attentive care. This memory became a hopeful vision for the kind of end-of-life experience she wished for others.

The theme *Finding Hope* underscores the interconnectedness of resilience and autonomy within the context of family dynamics. Engaging in future planning can empower care partners to feel more in control of their caregiving journey, instilling a sense of hope and optimism for the future. By actively preparing for potential challenges, some care partners experienced greater peace of mind and confidence in their ability to navigate the uncertainties of dementia caregiving.

## Discussion

Our study explored the experiences of care partners who provide ongoing care to persons living with dementia, capturing their insights through interviews and diary entries. We identified four key themes: (1) changes to living arrangements; (2) anticipatory grief; (3) future planning with changing health; and (4) finding hope. These themes underscore the complex emotional and practical challenges care partners face in planning for the future amidst the unpredictable trajectory of dementia.

Our findings resonate with existing literature on future planning for individuals with chronic illness, highlighting the complexity of navigating personal, interpersonal, financial, and systemic barriers [31, 32]. Despite the recognized importance of future planning, particularly for individuals who may experience diminishing decision-making capacity, it remains uncommon both formally and informally [33]. Similar to previous work [33], we found that effective future planning is predicated on factors such as living arrangements, health status changes, and the need to address the hopes and fears of both care partners and their relatives with dementia.

Our findings offer insights into the challenges of navigating shifting living arrangements, evolving health conditions, and the necessity to sustain hope in the context of future planning. Specifically, we found that future planning was inhibited by care partner burnout and concerns surrounding their own worsening health, as well as uncertainty regarding navigating a relative with dementia’s shifting capacity and decline. Research has shown that care partner burnout significantly impacts their ability to plan for the future and provides a barrier to accessing resources or making long-term decisions [34]. For instance, many care partners expressed anxiety about their ability to continue providing care as their own health deteriorated, and uncertainty about how to adapt to their relative’s changing needs over time. This concern is particularly common in dementia caregiving, where the unpredictability of the disease adds an additional layer of stress [35]. Care partners varied in their mental and emotional readiness for future planning, particularly regarding end-of-life considerations. This underscores the need for early conversations and comprehensive support that address not only the practical aspects of end-of-life and funeral planning but also the social, psychological, and emotional preparations. Such holistic support can alleviate the overwhelming burden that care partners often face when navigating these complex issues alone [36].

The majority of our study participants were women (75%), echoing prior research demonstrating their heightened involvement in caregiving and future planning activities compared to men [37]. Previous research suggests that women face distinct challenges in caregiving, often linked to their disproportionate engagement in socioemotional labor as part of care work [37]. Our study reveals that future planning constitutes a significant aspect of this labor, underscoring the need for further research on effective strategies to support women caregivers in this role [38, 39]. Future planning interventions, especially those focusing on end-of-life preferences and legacy arrangements (i.e., the distribution of one’s assets after death), should be designed to address the specific needs, preferences, and caregiving dynamics experienced by women. Additionally, future research should explore gender-specific experiences of future planning among women care partners to better understand how they can be effectively supported.

Our results further showed that effective future planning was facilitated by proactive modifications made to care partners’ homes and changes in living arrangements to manage relative with dementia’s changing abilities. Examples include installing grab bars, adjusting lighting, and creating more accessible living spaces, which have been shown to significantly improve safety and ease of care [40]. Educational and financial support are potential mechanisms to help care partners implement these safe home modifications and manage health changes effectively [41]. For example, providing informational modules on how to care for a person living with dementia and offering financial subsidies for home modifications can alleviate some of the physical and financial burdens on care partners [42].

### Theoretical implications: a new conceptual model of relational autonomy in the context of dementia-related future planning

**Fig. 1:**
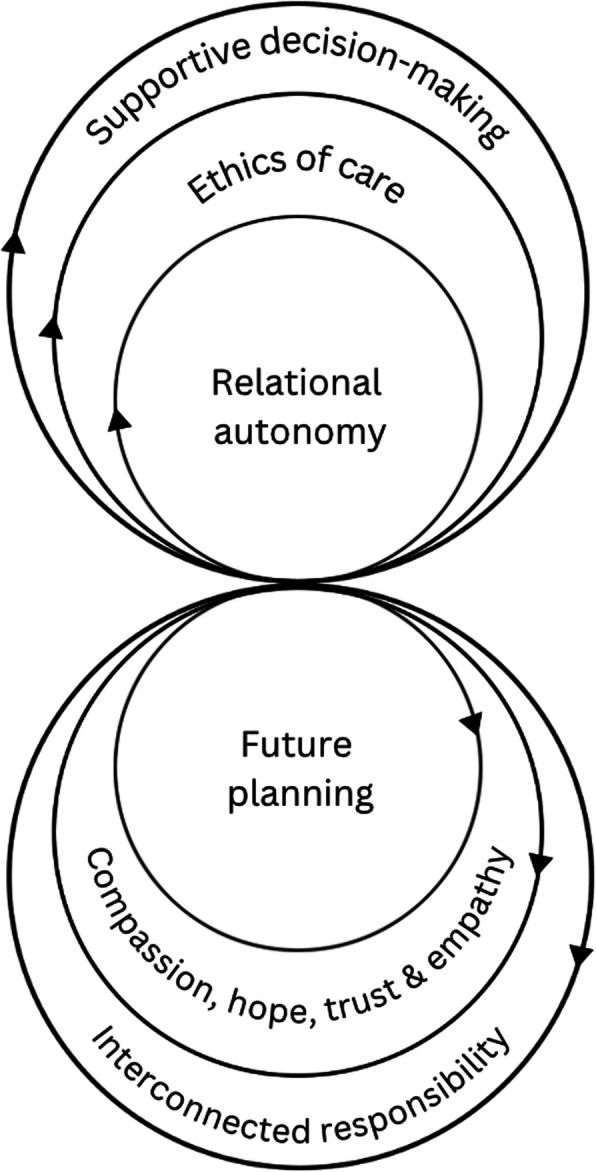
Conceptual model of relational autonomy in the context of dementia-related future planning

Our study has theoretical implications in the context of understanding and supporting future planning among persons caring for individuals with diminished capacity. The conceptual model in Fig. 1 illustrates the dynamic interplay between relational autonomy and future planning for persons living with dementia. At its core, relational autonomy, grounded in the ethics of care, emphasizes attentiveness, responsibility, competence, and responsiveness [19].

Each outer layer in the upper half of the model reinforces the core principles of relational autonomy. For example, supportive decision-making (i.e., enabling individuals with cognitive impairments to make informed decisions with guidance from care partners) facilitates autonomy by allowing individuals with dementia to actively participate in choices about their lives with the support of care partners, ultimately preserving dignity and self-agency. The ethics of care further underlines this approach, prioritizing compassionate and responsive care practices that recognize the individual as part of a network of relationships. In the lower half of the model, future planning is positioned as integral to relational autonomy. Effective future planning requires a foundation of interconnected responsibility, where care partners, healthcare providers, and social care professionals collaborate to anticipate and address the needs of individuals living with dementia as the disease progresses [13]. This responsibility is strengthened by compassion, hope, trust, and empathy, which foster a supportive environment for making future-oriented decisions that respect the values and well-being of the individual. The intertwined circles between relational autonomy and future planning illustrate the importance of a holistic approach. Ensuring dignity and autonomy requires not only ethical care and supportive decision-making but also a commitment to compassion, shared responsibility, and trust. This integrated approach aligns with findings that supporting the autonomy and future needs of individuals living with dementia calls for a multi-dimensional model that respects personhood and anticipates evolving care needs over time [43].

Previous research on care planning among families and persons living with dementia has drawn on other foundational concepts such as person-centred care [43, 44]. A relational autonomy approach to care extends beyond the tenets of person-centered care by recognizing that individuals’ autonomy is shaped by their relationships and social contexts [43]. While person-centered care focuses on respecting and addressing individual preferences and needs, relational autonomy emphasizes the interconnectedness of individuals and acknowledges how care partners, families, and broader societal structures influence decision-making and well-being [45]. This conceptual model offers a valuable foundation for future research and practice focused on supporting individuals living with dementia and their care partners in future planning. This model could guide studies aimed at identifying effective strategies that promote relational autonomy by examining how care partners and individuals living with dementia navigate future-oriented decisions within an ethics of care framework. It provides a structure for evaluating interventions that enhance supportive decision-making and dignity-preserving practices, allowing researchers to explore the impact of compassion, trust, and shared responsibility on the caregiving experience.

### Study limitations

Our sample was predominantly White women with a relatively high level of education and household income. Limited sample diversity means the experiences of other groups – such as persons who are members of sexual or cultural minority groups, with lower educational attainment, and/or with lower income levels – may not be fully represented in the findings. We drew on data collected as part of a larger study examining the experiences of family care partners across time and in relation to their interactions with the formal care system and negotiation of their caring role; the overarching goal of this project was not the investigation of future planning among families who are navigating a dementia diagnosis and disease trajectory. As such, there are aspects of future planning among this group that were likely not fully captured here. Data collection from participants concluded in the event the person living with dementia moved into a LTC home or passed away. Consequently, our findings cannot speak to future planning processes among care partners of persons living with dementia who are residing in LTC.

## Conclusions

This study provides valuable insights into the complex dynamics of future planning for care partners of individuals living with dementia. It reveals the profound impact of social relationships, the collaborative nature of decision-making, and the nuanced interplay between individual autonomy and evolving health conditions. Our research also demonstrates that relational autonomy offers a robust framework for understanding the interconnected nature of future planning in dementia care. This framework supports inclusive decision-making, acknowledges interdependence, respects personal relationships, and upholds ethical principles. Our findings underscore the need for educational initiatives targeted at both care partners and healthcare providers to enhance awareness and understanding of the importance of future planning. Additionally, the provision of accessible, comprehensive, and culturally competent information resources is essential to guide care partners through the future planning process for their relatives with dementia.

## Supplementary Information

Supplementary Material 1.

## Data Availability

The data that support the findings of this study are available from the senior author, Dr. Jennifer Baumbusch (jennifer.baumbusch@ubc.ca), upon reasonable request.

## Abbreviations

COVID-19: Coronavirus
LTC: Long-term care
