# Supplementary material for: Between hope and future planning: the dementia journey for care partners through the lens of relational autonomy
Source: BMC Med Ethics. 2025 Mar 21;26:39. doi: 10.1186/s12910-025-01197-2 (PMC11929221; doi:10.1186/s12910-025-01197-2)
Supplement: Supplementary file 1 — Supplementary Material 1. [file 12910_2025_1197_MOESM1_ESM.docx]

**Supplementary File 1. Interview guide.**

Code assigned to participant: _____

Code assigned to other member of dyad: _____

Date of Interview: ______________

1. Tell me about your dementia journey as a caregiver so far.
   1. When did you first notice the person you care for might have cognitive changes?
   2. What happened during the journey to a diagnosis? How was the diagnosis introduced? Who shared it with you?
   3. What types of services and supports did you access early in the dementia journey?
   4. What types of services and supports did you access before COVID-19 precautions (e.g. in the Fall of 2019)?
   5. Do you think your gender or the gender of the person you care for influenced this journey in any ways?
2. In addition to the support you provide your relative, did they have any other in-home supports in the Fall of 2019?
   1. Did they receive home support services (e.g. a staff person comes to your home to help with various activities)?
   2. Are in-home supports paid for by the health authority (with a client co-payment) or do you pay out-of-pocket? If you pay out-of-pocket, approximately how much do you pay per month?
   3. Did they attend any community programs, such as an adult day program, or respite?
   4. Do other relatives also help with care? If so, how?
   5. Do other members of your community help with care (e.g. neighbours, friends, church members)? If so, how?
   6. How have formal in-home or community-based supports changed since COVID-19 precautions came into effect?
3. Could you describe the kind of day-to-day help and support you provide for the person you care at home at this point in time, particularly in relation to their dementia?
   1. Alternative prompt: describe a typical day.
   2. Do you think your gender, or the gender of the person you care for has played a role in the type of care you provide to them?
4. Specifically in relation to COVID-19, have there been any government programs (federal, provincial) that you have found supportive?
   1. Are there any programs offered by local organizations that you have found helpful.
   2. Do you have recommendations for programs/services that would be helpful to you right now?
5. Is there anything else you’d like to tell me about your experiences as a caregiver for someone living with dementia?
